# Individualized Risk Prediction of Medical Postoperative Complications After Oncologic Hepatectomy: A Nomogram-Based Approach

**DOI:** 10.3390/medsci13040267

**Published:** 2025-11-13

**Authors:** Raluca Zaharia, Stefan Morarasu, Cristian Ene Roata, Ana Maria Musina, Wee Liam Ong, Gabriel Mihail Dimofte, Sorinel Lunca

**Affiliations:** 1 Grigore T Popa University of Medicine and Pharmacy, Iasi, Romania; raluca.zaharia11@yahoo.com (R.Z.); cristian.roata@umfiasi.ro (C.E.R.); ana-maria.musina@umfiasi.ro (A.M.M.); william05021990@gmail.com (W.L.O.); mihail.dimofte@umfiasi.ro (G.M.D.); sorinel.lunca@umfiasi.ro (S.L.); 2 2nd Department of Surgical Oncology, Regional Institute of Oncology, Iasi, Romania

**Keywords:** liver resection, postoperative complications, medical morbidity, surgical morbidity, hepatic surgery, outcomes, frailty

## Abstract

Background: Liver resection remains the primary curative treatment for many malignant liver diseases. Advances in patient selection, perioperative care, and surgical technique have markedly reduced procedure-related (surgical) complications in experienced centres. However, despite these improvements, medical (non-surgical) complications continue to represent a substantial source of postoperative morbidity, particularly after major liver resections. Herein, we aim to assess the incidence, nature, and predictors of medical versus surgical complications after liver resection and to develop an individual risk calculator for estimating medical morbidity after liver resection. Methods: This is an observational single-centre study including patients who underwent liver resection for cancer between 2013 and 2025. Postoperative complications were classified into medical and surgical categories based on clinical and diagnostic criteria. Demographic, clinical, and intraoperative data were analyzed to identify risk factors associated with each type of complication, and a multivariate logistic regression model was used to select significant variables, which were imputed in a prediction nomogram made available as an interactive web-based calculator. Results: Of the 231 patients included, 36 patients (15.6%) developed postoperative complications. From multivariate analysis, independent predictors of medical complications included cirrhosis (OR 2.8, 95% CI 1.2–6.8, *p* < 0.05), operative time > 180 min (OR 2.0, 95% CI 1.1–7.4, *p* < 0.05), intraoperative blood loss > 500 mL (OR 2, 95% CI: 0.9–4.8, *p* < 0.05), and ASA score ≥ 3 (OR 3.7, 95% CI 1.1–12.5, *p* < 0.05). Major hepatic resection was the only independent predictor of surgical complications (OR 7.42, 95% CI: 1.14–48.52, *p* = 0.036). The logistic regression model demonstrated fair discriminative ability with an AUC of 0.682 (95% CI: 0.544–0.729). The risk-prediction nomogram showed a 24.7% risk of postoperative medical morbidity in patients with all four risk factors vs. a 5.4% risk in patients without any risk factor. Conclusion: Postoperative medical complications are significantly more frequent in patients undergoing oncological liver resection with an ASA score ≥ 3, history of cirrhosis, prolonged operative time, and increased intraoperative blood loss. Our logistic regression model and web-friendly nomogram may be used for external validation in larger cohorts and could support preoperative counselling and perioperative risk stratification.

## 1. Introduction

Liver resection is a cornerstone of curative treatment for a wide spectrum of hepatic malignancies, including hepatocellular carcinoma (HCC), colorectal liver metastases (CRLM), and cholangiocarcinoma [1,2,3]. As surgical techniques, anesthetic care, and perioperative pathways have evolved, the safety and efficacy of hepatic resections have improved markedly, with perioperative mortality in experienced centres now falling below 5–7%. Nevertheless, the overall burden of postoperative complications remains substantial, affecting up to 40–50% of patients, and continues to significantly influence recovery trajectories, length of hospital stays, and early mortality [4,5].

Postoperative complications following hepatic resection are heterogeneous in nature and are typically categorized as either surgical—resulting directly from operative technique (e.g., bile leak, hemorrhage, intra-abdominal abscess)—or medical, referring to systemic decompensation or end-organ dysfunction (e.g., hepatic insufficiency, renal failure, respiratory distress, cardiac events, or sepsis). Historically, much of the surgical literature and clinical focus has centred on minimizing and managing surgical complications, such as bile leaks or intra-abdominal collections. However, this perspective may obscure a more pervasive and clinically significant issue: the high incidence and impact of non-surgical, medical complications [6,7,8,9,10,11].

Medical complications are increasingly recognized as a dominant contributor to adverse postoperative outcomes. These systemic events—including respiratory insufficiency, acute kidney injury, cardiovascular instability, hepatic decompensation, and sepsis—may arise not from technical mishaps but from the physiological stress of surgery superimposed on pre-existing organ dysfunction or systemic vulnerability. Importantly, such complications often demand intensive and multidisciplinary management, can lead to prolonged hospital stays or readmissions, and are frequently implicated in early postoperative mortality [12,13,14,15].

Despite these observations, the current body of literature lacks robust comparative data on the relative incidence and clinical burden of medical versus surgical complications following liver resection. Many studies tend to report complications in aggregate or focus on technical outcomes, thereby underestimating the prevalence and prognostic significance of medical morbidity. Furthermore, the existing classifications of postoperative complications often fail to distinguish clearly between surgical and medical events, limiting the ability to develop tailored prevention and intervention strategies [16,17].

In this context, our study seeks to address this gap by providing a detailed analysis of postoperative complications following liver resection, with an emphasis on distinguishing between surgical and medical aetiologies. Through retrospective evaluation of a well-defined institutional cohort, we aim to quantify the incidence of each complication type, explore associated patient and procedural risk factors, and assess their respective contributions to overall postoperative morbidity. By delineating these patterns, we intend to highlight the underappreciated burden of medical complications and advocate for a more holistic, patient-centred approach to perioperative risk assessment and management.

## 2. Materials and Methods

### 2.1. Design and Setting

This is a single-centre, single-department, single-surgeon, observational study of patients diagnosed with liver malignancies, both primary and secondary, based on medical history, imaging, or histopathological examinations. We retrospectively collected patients who underwent surgery at our institution between 2013 and 2025. Every patient received a conventional oncological work-up and multidisciplinary meeting-based care. All patients were treated and followed at our institution. Ethical approval and informed consent were waived for this study as the data was collected retrospectively, all patient data was anonymized and did not pose any risk to the patients.

### 2.2. Inclusion and Exclusion Criteria

**Figure 1:**
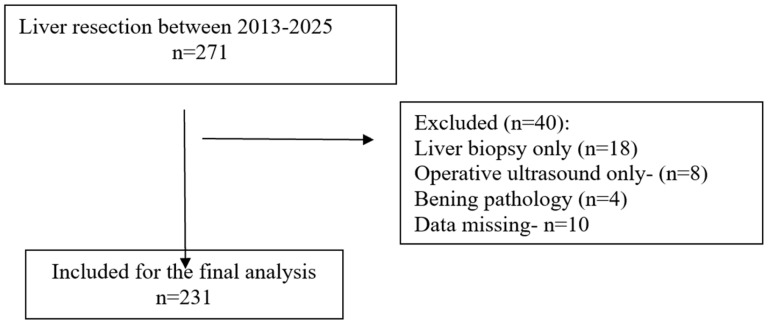
STROBE flow diagram.

The study was conducted in accordance with the STROBE checklist (Figure 1) [18]. All patients diagnosed with primary or secondary liver malignancies who underwent hepatic resection were eligible for inclusion. Only patients who underwent minor (resection of fewer than three hepatic segments) or major (resection of three or more segments) liver resections were included in the final analysis. Procedures limited to liver biopsy or intraoperative ultrasound without resection were excluded.

### 2.3. Data Extraction

Clinical, demographic, laboratory, and perioperative data were collected from institutional databases and electronic health records, including age, sex, comorbidities, and operative details. Preoperative laboratory tests and tumour-related factors were also recorded. A summary of these baseline characteristics is provided in Table 1. Postoperative complications were independently reviewed and classified into two main categories, medical and surgical, based on predefined clinical and diagnostic criteria derived from established hepatobiliary literature. Medical complications were defined as events unrelated to the surgical site (e.g., pulmonary, renal, cardiovascular, hepatic, metabolic, thromboembolic, infectious or septic events) while surgical complications were defined as events directly attributable to the surgical field, operative technique, or anastomotic failure, including but not limited to bile leaks, abdominal collections, hemorrhage, post hepatectomy liver failure, wound infection, postoperative ileus/obstruction. Each case was reviewed by two investigators independently, and discrepancies were resolved by consensus to minimize subjectivity.

### 2.4. Data Analysis

Continuous variables were expressed as means with standard deviations or as medians with interquartile ranges, according to distribution. Categorical variables were summarized as absolute frequencies and percentages. Group comparisons were performed using the chi-square test or Fisher’s exact test for categorical variables, and the independent samples *t*-test for continuous variables. To identify independent predictors of postoperative complications, multivariate logistic regression models were applied separately for medical and surgical outcomes. Due to the limited number of surgical complications, several predictors could not be reliably estimated, and instances of complete separation occurred, restricting the stability of the regression model. Statistical significance was set at *p* < 0.05. Significant variables were included in the final risk prediction model. The model was fitted using the lrm function from the rms package in R (version 4.5.1). Internal validation was conducted with 1000 bootstrap resamples, and model performance was expressed as the area under the receiver operating characteristic curve (AUC) and the optimism-corrected concordance index (C-index). Calibration was evaluated through slope and intercept estimation and graphically represented by a bias-corrected calibration plot. Based on the final regression model, we constructed a graphical nomogram to estimate the probability of medical complications. To facilitate clinical applicability, we further implemented the model as an interactive web-based risk calculator using Shiny for R, which allows clinicians to input patient characteristics and obtain individualized risk estimates.

## 3. Results

### 3.1. Patient Characteristics

A total of 231 patients were included (Figure 1), with a predominance of males (≈61%) and a mean age of 62.8 years. Most patients had low to intermediate frailty scores, while higher scores (≥2) were less frequent. The most common comorbidities were viral hepatitis (33%), cirrhosis (24%), and diabetes mellitus (21%), followed by chronic kidney disease and heart failure. According to the ASA classification, the majority were graded as ASA III (66%). Surgical indications were mainly hepatocellular carcinoma (42%) and liver metastases (51%), with a small proportion of cholangiocarcinoma. Preoperative laboratory values were overall within normal limits, although alterations consistent with underlying liver disease were present. Regarding operative characteristics, 17% of patients underwent major hepatectomy, nearly one-third presented with multiple lesions, and the mean operative time and intraoperative blood loss reflected moderate surgical complexity. A comprehensive summary of patient demographics, comorbidities, laboratory findings, and operative data is provided in Table 1 [13].

### 3.2. Incidence and Distribution of Postoperative Complications

Among the 231 patients, 36 (15.6%) developed postoperative complications. Medical events were more frequent than surgical ones, either occurring alone or in combination, and accounted for about three-quarters of all morbidity. By contrast, isolated surgical complications were less common. Most patients (over 80%) had an uncomplicated postoperative course. The detailed distribution of complication types is shown in Table 2.

### 3.3. Types of Postoperative Complications

Postoperative complications were identified in 36 of the 231 patients, corresponding to an overall morbidity rate of 15.6%. These events were classified into surgical and medical categories. Surgical complications were less frequent, consisting of intra-abdominal collections in 6 patients (2.6%), bleeding or hematoma in 4 (1.7%), bile leakage in 2 (0.9%), wound infection in 2 (0.9%), and bowel obstruction in 2 (0.9%).

Medical complications predominated, encompassing renal dysfunction in 9 patients (3.9%), pneumopathy in 8 (3.4%), cardiovascular events in 5 (2.1%), liver failure in 2 (0.9%), pulmonary embolism in 2 (0.9%), and a single occurrence of multiple organ dysfunction syndrome (0.4%) (Table 3).

### 3.4. Patient Profiles According to Complication Type

Patients with medical complications had a significantly higher mean age compared with those with surgical complications, while gender distribution and BMI did not differ between groups. An ASA score ≥ 3 was present in a similar proportion of cases in both categories. The rates of severe morbidity (Clavien–Dindo grade III–IV) were also comparable. A modified frailty index >2, as well as comorbidities including cirrhosis, diabetes mellitus, chronic obstructive pulmonary disease, and hypoalbuminemia, were recorded at similar frequencies, without statistically significant differences.

With respect to operative characteristics, major hepatic resections were more frequent in the surgical complication group, although the difference was not significant. Intraoperative blood loss > 500 mL was observed in both groups at comparable rates. Operative time > 180 min was recorded in all patients with surgical complications and in 75% of those with medical complications, representing the only variable that reached statistical significance. A detailed distribution of these characteristics is provided in Table 4.

### 3.5. Multivariate Analysis of Predictors for Medical Complications

Multivariate logistic regression was performed to identify independent predictors for postoperative medical complications after liver resection. The following variables were entered based on their clinical relevance and significance in univariate analyses: liver cirrhosis, diabetes mellitus, hypoalbuminemia (<3.5 g/dL), major hepatic resection, operative time > 180 min, intraoperative blood loss > 500 mL, and ASA score ≥ 3.

Several independent predictors for postoperative medical complications following liver resection were identified. Cirrhosis was associated with a more than two-fold increase in the odds of developing medical complications (OR 2.8, 95% CI 1.2–6.7, *p* < 0.05). Operative time > 180 min was also a significant factor, increasing the odds nearly three times (OR 2.8, 95% CI 1.1–7.4, *p* < 0.05). Intraoperative blood loss > 500 mL was correlated with approximately double the risk (OR 2, 95% CI 0.9–4.8, *p* < 0.05). The strongest association was observed for ASA score ≥ 3, which was linked to a more than three-fold increase in the likelihood of postoperative medical complications (OR 3.7, 95% CI 1.1–12.5, *p* < 0.05). Wide confidence intervals reflected the limited sample size and low number of events.

**Figure 2:**
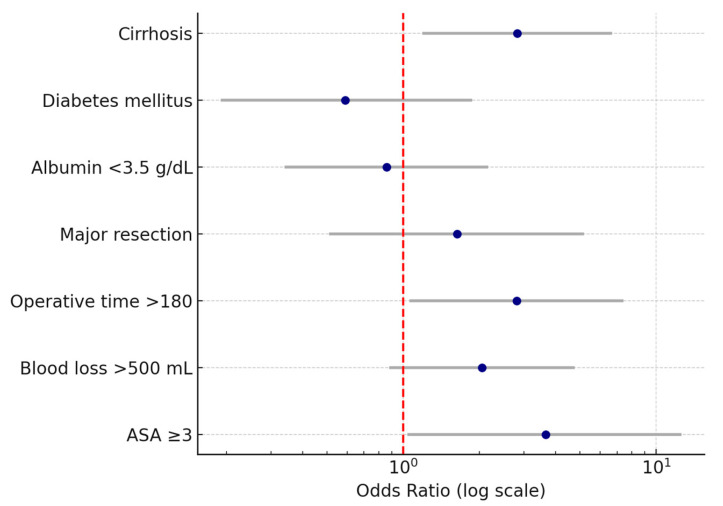
Forest plot of odds ratios (OR) and 95% confidence intervals for independent predictors of postoperative medical complications after liver resection. The vertical dashed line represents the null value (OR = 1). Note: Wide confidence intervals reflect variability related to sample size; only variables with statistically significant associations are interpreted as independent predictors.

In contrast, diabetes mellitus, hypoalbuminemia (<3.5 g/dL), and major hepatic resection did not retain statistical significance in the adjusted model, indicating that their effects observed in univariate analyses were likely influenced by coexisting factors. The results of the multivariate analysis are presented in Table 5 and Figure 2.

### 3.6. Individualized Risk Estimation Using the Nomogram Model

**Figure 3:**
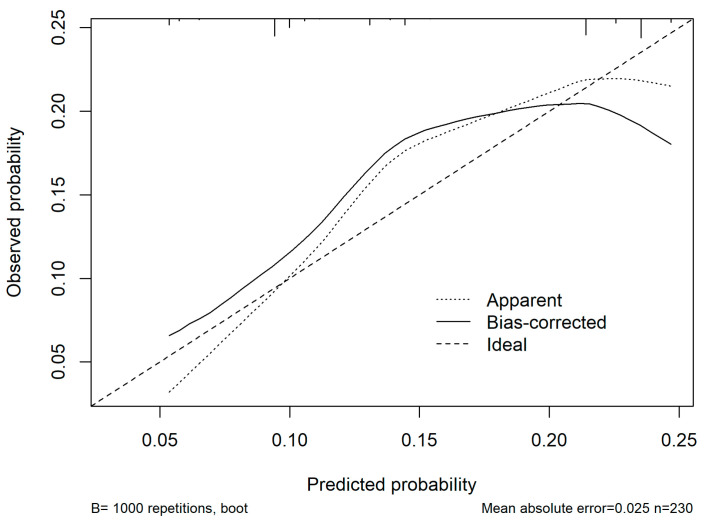
Calibration curve of the nomogram model for predicting postoperative medical complications. The *x*-axis represents predicted probabilities, and the *y*-axis represents the observed probabilities of complications. The dashed diagonal line indicates perfect calibration (ideal model), where predicted and observed risks are identical. The dotted line represents the apparent calibration of the model in the original dataset, while the solid line represents the bias-corrected performance after 1000 bootstrap resamples. The bias-corrected curve closely follows the ideal line across most probability ranges, indicating good agreement between predicted and observed risks with minimal overfitting (mean absolute error = 0.025).

In the multivariable analysis, ASA ≥ 3, cirrhosis, operative time > 180 min, and blood loss > 500 mL were all independently associated with the occurrence of medical complications. The logistic regression model demonstrated good discriminative ability with an AUC of 0.68 (95% CI: 0.5–0.7), and the optimism-corrected C-index after bootstrap validation was 0.6. Calibration analysis showed good agreement between predicted and observed risks across deciles of probability with a mean absolute error of 0.025 (Figure 3). Decision-curve analysis was considered but ultimately not performed due to the small sample size and limited event rate.

**Figure 4:**
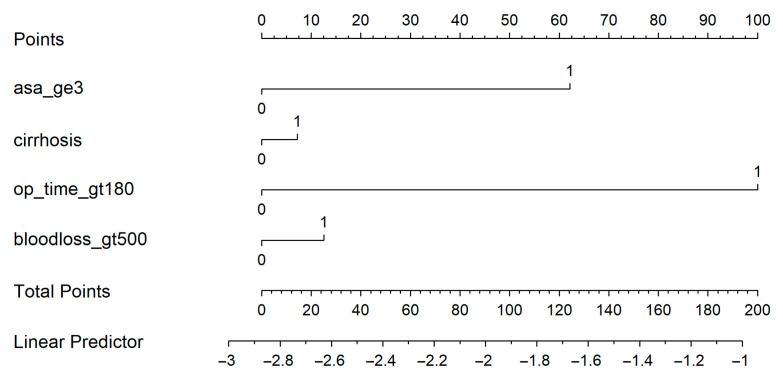
Nomogram predicts the probability of postoperative medical complications. Each variable contributes a weighted point value; the total score corresponds to the predicted risk shown on the bottom scale.

**Figure 5:**
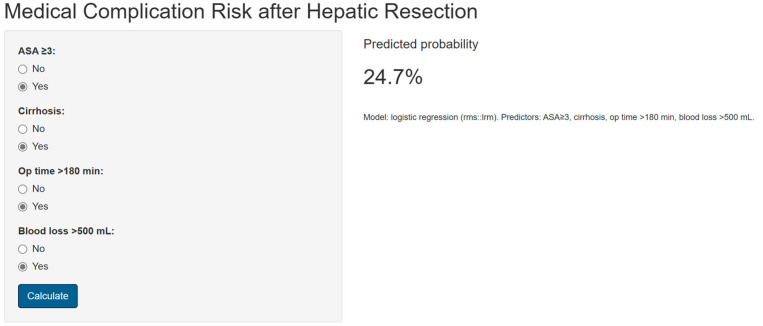
Snapshot showing the online available risk calculator (https://morarasustefan.shinyapps.io/hep_nomogram/, accessed on 25 October 2025).

A nomogram was generated based on the final model (Figure 4). Each predictor was assigned a weighted score, and the total score corresponds to the estimated probability of a postoperative medical complication. For instance, a cirrhotic patient with ASA ≥ 3, operative time > 180 min, and blood loss > 500 mL had an estimated complication risk of 24.7%. To enhance usability, we provide an interactive web-based calculator (Figure 5; full calculator available at: https://morarasustefan.shinyapps.io/hep_nomogram/, accessed on 25 October 2025), enabling individualized risk prediction in clinical practice. Wide confidence intervals reflected the limited sample size and low number of events. We therefore applied bootstrap internal validation with 1000 resamples to minimize optimism and correct the C-index. Multicollinearity was assessed (VIF < 2), confirming no significant interdependence between predictors.

## 4. Discussion

In this single-centre cohort of patients undergoing hepatic resection, we identified several key perioperative factors independently associated with the development of postoperative medical complications, including ASA score ≥ 3, presence of cirrhosis, prolonged operative time, and increased intraoperative blood loss. Based on these variables, we constructed a multivariable logistic regression model that demonstrated fair discriminative ability and good calibration after internal bootstrap validation. To enhance its clinical applicability, the model was translated into an easy-to-use nomogram and an interactive web-based calculator, allowing individualized risk estimation for each patient. This study provides clinicians with a practical tool to support preoperative counselling, perioperative planning, and identification of patients who may benefit from optimized management strategies.

Postoperative morbidity after liver resection continues to represent a major clinical concern, despite significant advances in surgical technique, anesthetic management, and perioperative care pathways [19,20]. Contemporary evidence indicates that adverse outcomes are not solely the consequence of technical errors or intraoperative surgical events but are largely determined by the interaction between patient comorbidities, physiological reserve, and intraoperative physiological stress [21,22]. Understanding the relative contributions of medical and surgical complications, as well as their underlying predictors, is therefore essential for improving perioperative management and optimizing patient outcomes [23,24,25,26,27].

In our cohort of 231 patients, postoperative morbidity occurred in 15.6% of cases. Medical complications predominated, representing three-quarters of all adverse events, while surgical complications were comparatively infrequent. Independent predictors of medical complications included ASA score ≥ 3, cirrhosis, and operative time exceeding 180 min, with intraoperative blood loss > 500 mL showing a non-significant trend toward increased risk. No independent predictors could be confirmed for surgical complications, reflecting the limited number of surgical events in our dataset and the instability of regression estimates for this subgroup. These findings highlight the systemic rather than purely technical determinants of morbidity in our population.

One of the most comprehensive analyses to date is the systematic review by Longchamp et al. (2021) [28], which synthesized data from 74 studies encompassing 68,480 patients undergoing liver resection. The authors reported that American Society of Anaesthesiologists (ASA) physical status score and intraoperative red blood cell transfusion were the two most consistent predictors of postoperative complications, with ASA associated with odds ratios ranging from 1.3 to 7.5, and transfusion with odds ratios from 1.2 to 17.1 [29]. Importantly, these predictors were significant across diverse patient populations, surgical extents, and indications for hepatectomy. This systematic review underscored the importance of preoperative global health status and intraoperative blood loss not only as markers of risk, but as potentially modifiable targets for intervention.

In a similar vein, Wang et al. (2014) [30] developed and validated a simple, integer-based risk score in 1057 patients with hepatitis B virus–related hepatocellular carcinoma. Their model, incorporating just four preoperative variables—ASA category, presence of portal hypertension, major hepatic resection, and concomitant extrahepatic procedures—stratified patients into low-, intermediate-, and high-risk categories, with predicted severe postoperative complication rates (Clavien–Dindo grade III–V) of 1.6%, 11.9%, and 65.6%, respectively. This work demonstrated that even a parsimonious set of clinically available variables could robustly differentiate postoperative risk, and it highlighted both ASA status and portal hypertension as core determinants of outcome.

More recently, Téoule et al. (2025) [31] analyzed a large contemporary cohort of patients undergoing laparoscopic liver resections, identifying ASA ≥ III, diabetes mellitus, and intraoperative packed red blood cell transfusion as independent predictors of serious complications (Clavien–Dindo ≥ III). Their study, which included both malignant and benign disease, confirmed that systemic comorbidities and intraoperative blood management play a pivotal role even in minimally invasive hepatic surgery.

Complementary evidence comes from Nagata et al. (2020) [32], who examined outcomes after open hepatectomy and reported that ASA class 3 (OR 11.5) and prolonged surgical duration were independently associated with major postoperative complications. This finding is particularly relevant to the present work, as it emphasizes the link between extended operative time, increased intraoperative stress, and postoperative morbidity—a connection that is often underappreciated when focusing solely on surgical site complications.

Taken together, these studies converge on a consistent set of risk factors—ASA status, presence of advanced liver disease (cirrhosis or portal hypertension), operative time, and intraoperative blood loss—that appear to transcend geographic regions, surgical approaches, and patient subgroups [4,33,34,35]. They also provide a strong conceptual basis for the findings of the present analysis.

Our study possesses several strengths, including its single-surgeon, single-centre design, which minimizes variability in operative technique and perioperative protocols. We also employed a clear classification of complications into medical and surgical categories, allowing for nuanced analysis of their respective predictors—a distinction often overlooked in the literature. An important strength of our study is the development of a user-friendly risk prediction tool. By transforming the multivariable model into both a nomogram and a web-based calculator, we provide clinicians with a practical means of estimating individualized risk of postoperative medical complications after hepatic resection. While this nomogram should not be used as a directive to limit or modify the type of oncologic liver resection, it may indeed facilitate preoperative counselling, shared decision-making, and optimization of perioperative care. For example, identifying high-risk patients prior to surgery may prompt closer monitoring, tailored anesthetic strategies, or enhanced recovery protocols. Importantly, the online calculator ensures accessibility and reproducibility, allowing clinicians and researchers to explore the model beyond the static representation in the manuscript. Our calculator can be used at the bedside and aid in patient preoperative counselling, especially in frail at-risk patients. It offers a clear, easy-to-understand, quantitative risk of medical complications that can occur after a major liver resection. The calculator takes into consideration the duration of surgery and blood loss, and this is important as patients can be informed that if surgery takes more than 3 h and more than 500 mL of blood is lost, then this directly impacts their recovery after surgery, despite having a well-performed operation with no technical incidents. On the other hand, ASA grade and history of cirrhosis are variables that are constantly available on the records of liver surgery candidates. Physicians can easily estimate postoperative risks just by taking a glimpse at their charts and at the ICU records, where operative time and blood loss are constantly documented in all countries. The ubiquity of these variables in any health system provides ease for multicentric studies and wide external validation of our model. While external validation in larger, independent cohorts is warranted, the availability of this tool represents an initial step towards personalized risk stratification in hepatobiliary surgery.

While operative time and blood loss are independent predictors when considering their influence in the logit regression model, from a clinical standpoint, they are merely surrogates, not direct risk factors for medical morbidity. Prolonged operative times illustrate a more difficult procedure, either from a surgical or anesthetic perspective. A difficult patient, either through their comorbidities/frailty or because of their complex tumour, will prolong operative time and thus will increase the rate of morbidity. While surgical complications can be prevented, to an extent, by careful dissection, haemostasis and reconstruction, medical complications cannot. Similarly, increased blood loss reflects a demanding case. However, with proper prehabilitation, operative planning and meticulous surgical and anesthetic technique, these factors can be modified, which will determine better postoperative outcomes.

Nevertheless, limitations must be acknowledged. The retrospective design inherently carries risks of selection and information bias. The relatively small number of surgical complications constrained the multivariable analysis for that subgroup, as reflected in the wide confidence intervals and non-estimable results. The inclusion of seven predictors with only 36 outcome events corresponds to approximately five events per variable, below conventional recommendations. We acknowledge this limitation explicitly and justify variable inclusion based on both clinical relevance and univariate associations. Penalized regression or shrinkage techniques, such as ridge or lasso, could further stabilize coefficients, and these will be considered in future external validations. Additionally, we did not systematically assess frailty, sarcopenia, or certain laboratory markers (e.g., prealbumin, inflammatory cytokines) that may further refine risk prediction. Also, cirrhosis was globally included, without stratification based on severity, nor did we include other scoring systems (e.g., MELD, ALBI) due to scarce availability in the records. Future research should focus on integrating multicentre cohorts and advanced modelling approaches (e.g., penalized logistic regression, gradient boosting, or random forest models) to verify whether nonlinear or interaction effects can improve discrimination without compromising interpretability. Decision-curve analysis will also be incorporated in subsequent studies to assess clinical utility across thresholds of risk tolerance.

## 5. Conclusions

In this single-centre analysis of patients undergoing hepatic resection, postoperative medical complications were independently associated with an ASA score ≥ 3, presence of cirrhosis, prolonged operative time, and increased intraoperative blood loss. The resulting multivariable model demonstrated fair discriminative ability and good calibration after internal bootstrap validation. Based on this model, a practical nomogram and an interactive web-based calculator were developed to facilitate individualized risk estimation. Although external validation in larger and more diverse cohorts is warranted, this tool may support preoperative counselling, perioperative risk stratification, and optimization of patient care in hepatobiliary surgery.

## Figures and Tables

**Table 1:** Patients’ Characteristics.

	n (%)
Total	231
Gender (males)	140 (60.6)
Age, mean (SD)	62.8
BMI	26.43 ± 0.3
mFI	
0	92 (38.8)
1	76 (32)
2	46 (19.4)
3	15 (6.3)
4	2 (0.8)
Comorbidities	
Viral hepatitis	79 (33.3)
Cirrhosis	57 (24)
Diabetes	49 (20.6)
CHF	31 (13)
COPD	20 (8.4)
CKD	37 (15.6)
Smoking	67 (28.2)
Neoadjuvant RT	5 (2.1)
Neoadjuvant CHT	55 (23.2)
Neoadjuvant RCHT	29 (12.2)
ASA 2	52 (21.9)
ASA 3	156 (65.8)
Hepatocellular carcinoma	96 (41.5)
Metastases	121 (51)
Cholangiocarcinoma	14 (6)
Preoperative blood tests	
Total bilirubin (mg/dL)	0.89 ± 0.9
Albumin (g/dL)	4.14 ± 0.8
INR	1.49 ± 0.8
PLT (mmc) × 10^3^	215.9 ± 6,2
AST	62.9 ± 17.1
ALT	52.8 ± 14.9
Operative factors	
Major resection	39 (16.1)
No. of liver lesions (≥2)	68 (28.7)
Operative time (min)	234.5 ± 12.2
Blood loss (mL)	548.7 ± 57.3

Key. mFI, modified frailty index; BMI, body mass index; CHF, Congestive Heart Failure; COPD, Chronic Obstructive Pulmonary Disease; CKD, Chronic Kidney Disease; ASA, American Society of Anesthesiologists; RT, radiotherapy; CHT, chemotherapy; RCHT, radiochemotherapy; INR, International Normalized Ratio; PLT, Platelets; AST, Aspartate Aminotransferase; ALT, Alanine Aminotransferase.

**Table 2:** Distribution of postoperative complications.

Complication Type	n	(%)
Medical	20	8.6
Surgical	9	3.9
Medical + Surgical	7	3
No complications	195	84.4

**Table 3:** Postoperative outcomes.

Total	n = 231 (100%)
Morbidity	n = 36 (15.6%)
Surgical complications	
Bile leaks	2 (0.9)
Bleeding/hematoma	4 (1.7)
Intra-abdominal collection	6 (2.6)
Wound infection	2 (0.9)
Bowel obstruction	2 (0.9)
Medical complications	
Liver failure	2 (0.9)
Pulmonary embolism	2 (0.9)
Pneumopathy	8 (3.4)
Cardiovascular	5 (2.1)
Renal	9 (3.9)
Mods	1 (4.3)

**Table 4:** Patient Profiles According to Complication Type.

	Medical Complications n (%) 20	Surgical Complications n (%) 9	Medical and Surgical Complications n (%) 7	No Complications n (%) 195	*p*-Value Medical vs. Surgical
Age, mean (SD)	66.6 ± 3.2	53.5 ± 3	74.2 ± 4.2	62.37 ± 1.4	<0.05
Gender Males	11 (55)	5 (55.6)	6 (85.7)	122 (62.6)	1
BMI, mean (SD)	27.6 ± 1.7	26.5 ± 3.3	26.7 ±2.3	26.45 ± 0.6	0.2
ASA ≥ 3 Clavien-Dindo III-IV mFi > 2	15 (75) 10 (50) 4 (1.7)	7 (77.8) 4 (44.4) 2 (0.9)	6 (85.7) 4 (44.4) 3 (1.3)	143 (73.3) 1 (57.1) 54 (23.4)	1 1 1
Cirrhosis	5 (25)	1 (11.1)	2 (11.1)	48 (28.6)	0.6
Diabetes mellitus	3 (15)	3 (33.3)	2 (33.3)	45 (28.6)	0.3
COPD	2 (10)	1 (11)	1 (11.1)	16 (14.3)	1
Low albumin (<3.5 g/dL)	6 (30)	4 (44.4)	2 (44.4)	49 (28.6)	0.6
Major resection	7 (35)	5 (55.6)	1 (55.6)	30 (14.3)	0.4
Operative time >180 (min)	15 (75)	9 (100)	4 (57.1)	107 (54.9)	<0.05
Blood loss > 500 mL LOS	11 (55) 8 ± 2.15 (±26.9%)	4 (44.4) 6.77 ± 1.5 (±26%)	1 (14.3) 9.57 ± 3.2 (±33.8%)	64 (32.8) 5.42 ± 0.3 (±13.5%)	0.6 <0.05
Blood transfusion	8 (40)	3 (33.3)	1 (14.2)	25 (12.8)	0.6

Key. SD, standard deviation; BMI, body mass index; ASA, American Society of Anesthesiologists; mFI, modified frailty index; COPD, Chronic Obstructive Pulmonary Disease; LOS, Length of Stay.

**Table 5:** Multivariate analysis of independent predictors for postoperative medical complications after liver resection.

Independent Variable	OR (Odds Ratio)	95% CI	*p*-Value
Cirrhosis	2.8	1.2–6.7	<0.05
Diabetes mellitus	0.6	0.2–1.9	0.36
Albumin < 3.5 g/dL	0.8	0.3–2.2	0.75
Major resection	1.6	0.51–5.2	0.40
Operative time > 180	2.8	1.1–7.4	<0.05
Blood loss > 500 mL	2	0.9–4.8	<0.05
ASA ≥ 3	3.7	1.1–12.5	<0.05

## Data Availability

The raw data supporting the conclusions of this article will be made available by the authors on request. Data cannot be made publicly available due to institutional ownership restrictions.

## Abbreviations

The following abbreviations are used in this manuscript:

AUC	Area Under the Curve
ASA	American Society of Anesthesiologists
ALT	Alanine Aminotransferase
AST	Aspartate Aminotransferase
BMI	Body Mass Index
CHT	Chemotherapy
CHF	Congestive Heart Failure
CI	Confidence Interval
CKD	Chronic Kidney Disease
COPD	Chronic Obstructive Pulmonary Disease
CRLM	Colorectal Liver Metastases
C-index	Concordance Index
HCC	Hepatocellular Carcinoma
INR	International Normalized Ratio
LOS	Length of Stay
mFI	Modified Frailty Index
MODS	Multiple Organ Dysfunction Syndrome
OR	Odds Ratio
PLT	Platelets
RCHT	Radiochemotherapy
RT	Radiotherapy
SD	Standard Deviation
STROBE	Strengthening the Reporting of Observational Studies in Epidemiology
